# Are the current MRI criteria using the DWI-FLAIR mismatch concept for selection of patients with wake-up stroke to thrombolysis excluding too many patients?

**DOI:** 10.1186/s13049-015-0101-7

**Published:** 2015-02-19

**Authors:** Audun Odland, Pål Særvoll, Rajiv Advani, Martin W Kurz, Kathinka D Kurz

**Affiliations:** Department of Radiology, Stavanger University Hospital, Postboks 8100, 4068 Stavanger, Norway; Faculty of Medicine, University of Oslo, Oslo, Norway; Department of Neurology, Stavanger University Hospital, Stavanger, Norway; Neuroscience Research Group, Stavanger University Hospital, Stavanger, Norway

**Keywords:** Brain, Diagnosis, Magnetic resonance imaging (MRI), Acute ischemic stroke, Fluid attenuated inversion recovery (FLAIR), Diffusion weighted imaging (DWI), Mismatch

## Abstract

**Background:**

Up to 25% of stroke patients wake up with a neurological deficit, so called wake-up stroke (WUS). Different imaging approaches that may aid in the selection of patients likely to benefit from reperfusion therapy are currently under investigation. The magnetic resonance imaging (MRI) diffusion weighted imaging – fluid attenuated inversion recovery (DWI-FLAIR) mismatch concept is one proposed method for identifying patients presenting within 4.5 hours of the ischemic event.

**Purpose:**

To report our experience with the DWI-FLAIR mismatch concept for selection of wake-up stroke patients to be thrombolysed at our centre.

**Material and methods:**

Patients treated with off label intravenous thrombolysis (IVT) for WUS at our centre during a 6.5-month period were included. We performed MRI including DWI and FLAIR in all patients at admission. Each MRI examination was rated as either DWI-FLAIR mismatch or match. National Institutes of Health Stroke Scale (NIHSS) and modified Rankin Scale were used to measure clinical outcome. Cerebral computed tomography (CT) or MRI was performed within 24 hours after thrombolysis to determine the presence of any intracranial haemorrhage (ICH).

**Results:**

Ten patients treated with IVT for WUS were included. Four patients had a DWI-FLAIR mismatch and after IVT treatment the mean reduction in NIHSS in the DWI-FLAIR mismatch group was 4.0. In the DWI-FLAIR match group the mean reduction in NIHSS after IVT therapy was 4.8. None of the ten patients had any signs of ICH on follow-up imaging.

**Conclusions:**

In this small series DWI-FLAIR mismatch was not associated with worse outcome or ICH. This suggests that selecting WUS patients using DWI-FLAIR mismatch in clinical trials may exclude a large group of patients who might benefit.

## Background

Results from large randomized controlled trials have shown a clear benefit for intravenous thrombolysis (IVT) when patients with ischemic stroke are treated within 4.5 hours (hrs) of symptom onset [1,2]. If IVT is administered later than 4.5 hrs after symptom onset, the risk of harm exceeds potential benefit [3]. In a large subgroup of patients with ischemic stroke the exact time of symptom onset is unknown. Up to 25% of patients wake from sleep with a neurological deficit; a so called wake-up stroke (WUS) [4,5]. It is still unclear if these patients benefit from thrombolytic therapy [6]. Several studies suggest that the majority of these strokes occur close to the time of awakening [7-9]. According to current guidelines, symptom onset is defined as the last documented time the patient was known to be asymptomatic [10]. As a result, WUS patients are precluded from receiving IVT treatment.

Different imaging modalities that may aid in patient selection for reperfusion therapy are currently under investigation. Besides the traditional non-enhanced computed tomography (CT) and perfusion-CT, two different magnetic resonance imaging (MRI) techniques are discussed [11]. The diffusion weighted imaging-fluid attenuated inversion recovery (DWI-FLAIR) mismatch concept indirectly estimates time of the ischemic event [12]. Penumbral imaging estimates the volume of hypoperfused tissue which is not irreversibly damaged, using DWI and perfusion imaging [13]. At our centre (Stavanger University Hospital), the MRI protocol for WUS patients includes both DWI and FLAIR series. However, the DWI-FLAIR mismatch concept is not rigorously used to exclude patients from IVT. The DWI-FLAIR mismatch concept has been shown to identify patients presenting within 4.5 hrs of symptom onset with a high positive predictive value (83–87%) [12,14]. Nonetheless, we believe that too many WUS patients are excluded from the benefits of IVT when strictly adhering to the mismatch concept. Subsequently, we present here our experience with the DWI-FLAIR mismatch concept in these patients.

## Material and methods

### Patient selection

We retrospectively identified WUS patients treated with off label IVT at Stavanger University Hospital between 06.11.2013 and 22.05.2014. All consecutive wake up patients who were eligible for intravenous thrombolysis were included. The WUS patients met the following criteria: (a) asymptomatic <12 but >4.5 hrs from onset; (b) patients could report that they woke with stroke symptoms or were seen to have deficits on awakening; (c) diffusion restriction in less than 1/3 of the middle cerebral artery territory on DWI; (d) fulfilling all other IVT treatment criteria. There were no patients eligible for thrombolysis and considered to have WUS who did not get treatment, neither because of contraindications for MRI nor contraindications for thrombolysis found on MR or CT images; such as haemorrhages, restricted diffusion in more than 1/3 of the middle cerebral artery territory on DWI or intracranial tumours found.

### Radiological imaging

All patients underwent an initial cerebral MRI. The MRI protocol comprised transversal DWI (b-factor 0 and 1000), transversal T2-weighted imaging, transversal FLAIR imaging, transversal T2* weighted imaging and transversal time of flight arterial MR angiography; representing a total scan time of 15 minutes. To save time, MR angiography was not performed in one patient (patient 5) who had undergone a CT examination with CT perfusion and CT angiography of precerebral and intracranial arteries prior to the MRI. All MR scans were performed on 1.5 Tesla MRI machines (GE Discovery 450, Philips Intera, Philips Ingenia). Follow up imaging with unenhanced CT or MRI was performed within 24 hours after IVT treatment to verify infarction, any eventual haemorrhagic complications and evaluate prognosis.

### Imaging interpretation and the DWI-FLAIR mismatch concept

**Figure 1:**
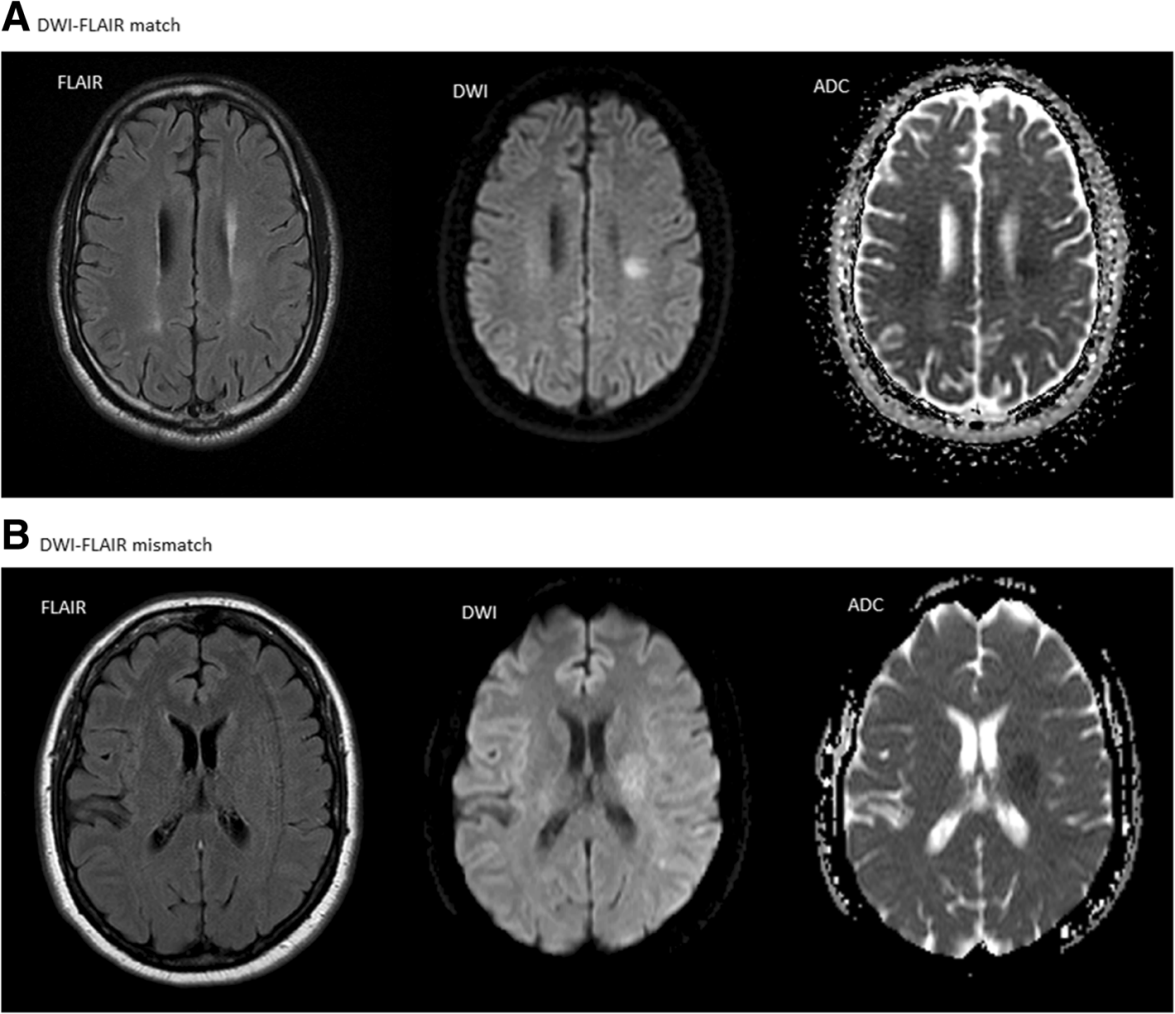
**Summary of data from the ten included patients.** Example of diffusion weighted imaging – fluid attenuated inversion recovery (DWI-FLAIR) match **(A)** and mismatch **(B)**. The DWI images are with b-value 1000. The apparent diffusion coefficient (ADC) maps are also displayed. The FLAIR image in B also illustrates the common problem with motion artifacts in these patients. The images in A are from patient 5 and the images in B from patient 6.

Diffusion restriction (positive DWI) was defined as increased signal on DWI b 1000, and corresponding reduced apparent diffusion coefficient (ADC) values. Increased signal on FLAIR images in the same area as the restricted diffusion was defined as positive FLAIR, whilst a normal signal on the FLAIR images was defined as negative FLAIR. DWI-FLAIR mismatch (Figure 1B) required positive DWI and negative FLAIR, as defined in previous studies [12,14-16]. DWI-FLAIR match was consequently defined as a positive DWI and a positive FLAIR (Figure 1A). In concordance with the study of Thomalla and coauthors, FLAIR was scored as positive even if only small areas of high signal on FLAIR images were identified within larger areas of restricted diffusion [12]. We did not use DWI-FLAIR match as strict exclusion criteria for IVT treatment. For study purposes 3 radiologists re-evaluated the initial MRI examinations performed on admission and reached a consensus on the DWI-FLAIR evaluation. The size of the largest positive DWI lesion in each patient was defined as the largest transversal diameter.

### Safety evaluation

The presence of intracranial haemorrhage (ICH) on post treatment CT or MRI was recorded. ICH was classified as in the European Cooperative Acute Stroke Study II (ECASS II) [17].

### Clinical evaluation

Neurologic deficit was graded on admission and at discharge using the National Institutes of Health Stroke Scale (NIHSS) [18-20]. The modified Rankin Scale (mRS) was used to assess the degree of disability or dependence in the daily activities [21]. The patients were retrospectively assessed for their mRS prior to the ischemic stroke and then assessed three months after.

### Ethics

Patients were prospectively collected and only included in this study after informed written consent was given. After retrospective analysis of the results, we now include our WUS patients in a nationwide, prospective study (NOR-TEST) to enhance the number of WUS available for analysis. The study was approved by the Regional Ethics Committee.

## Results

A total of ten patients presenting with WUS were treated with off-label IVT during the study period (Table 1). The mean age of the patients was 66 years (range 33 – 90 years). Most ischemic lesions were small, five patients presenting with DWI lesions < 15 mm. One patient had an infratentorial lesion (patient 7) and one patient (patient 8) had ischemic lesions in the posterior cerebral artery territory. Despite the ischemic lesions on DWI being small, several patients had significant neurologic deficits; e.g. patient 2, with multiple small DWI lesions in the watershed area following the occlusion of one branch of the M1 segment of the right middle cerebral artery (MCA). No patients suffered from intracranial haemorrhage after reperfusion therapy (ECASS II grade 0).

**Table 1:** **Summary of results from the ten included patients**

**Patient**	**Age**	**NIHSS on admission**	**Size of largest DWI lesion**	**DWI-FLAIR**	**Therapy**	**NIHSS at discharge**	**mRS before stroke and at 3 months**	**MRA/CTA**
**1**	86	5	16 mm	Match	IVT	5	3 - 4	M1 stenosis
**2**	68	8	10 mm	Match	IVT	2	0 - 1	M1 branch occlusion Improved FU
**3**	53	5	12 mm	Match	IVT	0	0 - 0	Normal
**4**	33	9	58 mm	Match	IVT and EVT	0	0 - 1	M1 occlusion Normal FU
**5**	64	8	22 mm	Match	IVT	1	0 - 0	CTA normal
**6**	51	19	26 mm	Mismatch	IVT and EVT	11	0 - 2	ICA MCA occlusion Normal FU
**7**	79	3	13 mm	Mismatch	IVT	1	2 - 2	Normal
**8**	90	3	7 mm	Match	IVT	1	3 - 3	P1 occlusion no FU
**9**	70	3	8 mm	Mismatch	IVT	0	0 - 2	Normal
**10**	65	4	16 mm	Mismatch	IVT	1	4 - 4	Stenosis M1, no FU

NIHSS: National Institutes of Health Stroke Scale. DWI: Diffusion Weighted Imaging. FLAIR: Fluid Attenuated Inversion Recovery. IVT: Intravenous thrombolysis. EVT: Endovascular thrombectomy. mRS: Modified Rank scale. MRA: MR angiography of the intracranial arteries in time of flight (TOF) technique. CTA: Computed tomography angiography of the precerebral and intracranial arteries. M1: M1 segment of the middle cerebral artery. P1: P1 segment of the posterior cerebral artery. ICA: Internal carotid artery. FU: Follow up CT or MR examination.

Follow up imaging with CT or MRI was performed 24 hrs after IVT treatment in nine patients. In one patient (patient 10) the follow up imaging was performed 10 hrs after IVT treatment.

Four patients had a clear DWI-FLAIR mismatch (patient 6, 7, 9 and 10). They received IVT treatment and had a reduction in NIHSS of 4.0 on average (range 8 – 2). None of these patients had signs of ICH on follow up imaging with CT or MRI.

Four patients had a clear DWI-FLAIR match (patient 3, 4, 5 and 8). In two patients, classification into DWI-FLAIR mismatch or match group was challenging. The first patient (patient 1) with extensive confluating hyperintense white matter lesions on FLAIR images presented with an area of positive DWI in the same area. There was a definite DWI-FLAIR match, but it is possible that the increased signal on the FLAIR images was a result of chronic and not acute ischemia in that area. This case was classified as DWI-FLAIR match. Another patient (patient 2) presenting with multiple transitory ischemic attacks (TIAs) within the preceding 24 hrs had multiple small (3–10 mm) lesions with positive DWI. Some of these, mainly the largest, also had a hyperintense signal on the FLAIR images in the corresponding area. However, it was not possible to decide which lesion was responsible for the acute clinical symptoms. This patient was also classified as DWI-FLAIR match. The patients with a DWI-FLAIR match received IVT treatment and had a mean reduction in NIHSS of 4.8 (range 9 – 0) (Table 1). Two patients, number 4 and 6, received additional endovascular treatment. None of the patients with a DWI-FLAIR match showed ICH on follow up imaging with CT (patient 1, 3, 5 and 8) and MRI (patient 2 and 4).

On time of flight intracranial MR angiography (MRA) three patients had normal findings, one patient had a M1 occlusion, one patient an occluded branch of the MCA, two patients showed stenoses in the M1 segment with reduced flow signal distal to the stenosis, one patient showed a P1 occlusion and one patient had a tandem occlusion in the internal carotid artery (ICA) and MCA. Follow up MR examination was not performed in all patients, however, one of the M1 occlusions (patient 4), the MCA branch occlusion (patient 2) and the tandem occlusion (patient 6) all showed restored flow signal on the MRA classified as TICI 3.

Three patients (3/6 = 50%) in the DWI-FLAIR match group showed a drop in mRS of one point each at three months compared to their mRS before the stroke. In the mismatch group two patients (2/4 = 50%) showed a drop in mRS of two points each. One patient (patient 10) was severely disabled (mRS 4) before the stroke event. This was a 65 years old male with bilateral blindness and mild cognitive impairment.

## Discussion

Although the presented cohort of ten patients is too small to draw general conclusions, we can show that six of the ten patients (60%) would have been excluded from IVT treatment if strict DWI-FLAIR mismatch criteria would have been adhered to [12]. All patients in this study received IVT treatment, yet only four patients had an indisputable DWI-FLAIR mismatch. No ICH was seen in either of the patient groups on follow up imaging; DWI-FLAIR mismatch versus DWI-FLAIR match. Both patient groups had similar improvements in NIHSS: 4 versus 4.8 respectively. The safety of IVT treatment in WUS patients without absolute contraindications on initial imaging was also confirmed in a recent study by Manawadu and colleagues [22]. Their study compared 68 WUS patients treated with IVT without any or only early ischemic changes on initial CT-imaging alongside 326 patients treated with IVT within 4.5 hrs. Their results, in congruence with ours, show that both groups profited equally (4 point improvement in NIHSS) with the same incidence of ICH (22% versus 20%, respectively) and symptomatic ICH (sICH) (2.9% versus 3.4%, respectively) on follow up imaging. These results highlight that IVT treatment is a beneficial therapeutic modality in acute stroke patients treated within 4.5 hrs [10]. Additionally, the authors conclude that IVT treatment seems to be a safe and feasible option in WUS patients without radiological contraindications [22,23].

The introduction of the DWI-FLAIR mismatch concept for identification of WUS patients within 4,5 hrs of symptom onset is clearly advantageous [12,14]. In the study by Thomalla et al. DWI-FLAIR mismatch identified patients within 4.5 hrs of symptom onset with a positive predictive value (PPV) of 0.83, a negative predictive value (NPV) of 0.54, sensitivity of 0.62 and specificity of 0.78 [12]. Lesion size was identified as a confounding factor due to the fact that this method is less suitable for smaller lesions; also verified by another study [16]. A similar study by Aoki et al. identified DWI-FLAIR mismatch patients within 4.5 hrs of symptom onset with a PPV of 0.87, NPV of 0.70, sensitivity of 0.74 and specificity of 0.85; this study excluded patients with lacunar infarctions (<15 mm), infratentorial infarctions or severe leukaraiosis [14].

Both the afore mentioned studies have a high PPV, suggesting that WUS patients with a larger ischemic lesion and DWI-FLAIR mismatch are in fact highly likely to be within 4.5 hours of symptom onset. However, the study by Thomalla et al. has a relatively low NPV (0.54) highlighting that many patients presenting within 4.5 hrs already have a DWI-FLAIR match [12]. Rigorous use of the DWI-FLAIR mismatch concept would exclude these patients from IVT treatment.

Ongoing trials selecting patients either with WUS (WAKE-UP trial) or beyond the 4.5 time window (EXTEND trial) based on identification of significant salvageable brain tissue may further contribute to better select patients profiting from IVT [24,25].

Our study has its limitations: The sample size is too low to draw any firm conclusions and the retrospective nature of the study is a clear disadvantage. We see that the infarct size in our study is on average small. Despite the DWI lesions being small, several patients showed large vessel occlusions on MRA, and one would therefore expect that some of the ischemic lesions seen on DWI would progress if not thrombolysed. We can add to the body of evidence that the DWI-FLAIR mismatch concept is just a stepping stone to other, more reliable imaging techniques. Techniques such as multiparametric CT and MRI may have the advantage of estimating penumbra more precisely and giving us a more reliable indication of salvageable brain tissue [26]. However, optimal methods for evaluating penumbra are still under investigation [11].

In this small series DWI-FLAIR mismatch was not associated with worse outcome or ICH. This suggests that selecting WUS patients using DWI-FLAIR mismatch in clinical trials may exclude a large group of patients who might benefit. Clearly more research is warranted to lead to advances in this field.

## Abbreviations

ADC: Apparent diffusion coefficient
CT: Computed tomography
DWI: Diffusion weighted imaging
ECASS II: European Cooperative Acute Stroke Study II
FLAIR: Fluid attenuated inversion recovery
Hrs: Hours
ICH: Intracranial haemorrhage
IVT: Intravenous thrombolysis
MCA: Middle cerebral artery
MRA: MR angiography
MRI: Magnetic resonance imaging
NIHSS: National Institutes of Health Stroke Scale
NPV: Negative predictive value
PPV: Positive predictive value
sICH: Symptomatic intracranial haemorrhage
TIA: Transitory ischemic attacks
WUS: Wake-up stroke
